# Hydroxytakakiamide and Other Constituents from a Marine Sponge-Associated Fungus *Aspergillus fischeri* MMERU23, and Antinociceptive Activity of Ergosterol Acetate, Acetylaszonalenin and Helvolic Acid

**DOI:** 10.3390/md22030097

**Published:** 2024-02-20

**Authors:** Harol Ricardo Arias Cardona, Bruno Cerqueira da Silva, Flávia Oliveira de Lima, Franco Henrique Andrade Leite, Bruno Cruz de Souza, Hugo Neves Brandão, Jorge Maurício David, Clayton Queiroz Alves, Anake Kijjoa

**Affiliations:** 1 Departamento de Saúde, Universidade Estadual Feira de Santana, Av. Transnordestina, Feira de Santana 3400-Papagaio, Bahia, Brazil; hrariasc@unal.edu.co (H.R.A.C.); farma.bruno@yahoo.com.br (B.C.d.S.); flavia_lima2000@yahoo.com.br (F.O.d.L.); fhaleite@uefs.br (F.H.A.L.); bcfarma@gmail.com (B.C.d.S.); hugo@uefs.br (H.N.B.); 2 Instituto de Química, Universidade Federal da Bahia, Av. Milton Santos, s/nº-Ondina, Salvador 40170-110, Bahia, Brazil; jmdavid@ufba.br; 3 Departamento de Ciências Exatas, Universidade Estadual Feira de Santana, Av. Transnordestina, Feira de Santana 3400-Papagaio, Bahia, Brazil; 4 ICBAS-Instituto de Ciências Biomédicas Abel Salazar, Universidade do Porto, Rua de Jorge Viterbo Ferreira, 228, 4050-313 Porto, Portugal

**Keywords:** *Aspergillus fischeri*, Aspergillaceae, marine sponge-associated fungus, hydroxytakakiamide, ergosterol acetate, acetylaszonalenin, helvolic acid, antinociceptive activity

## Abstract

An unreported prenylated indole derivative hydroxytakakiamide (**4**) was isolated, together with the previously described ergosterol (**1**), ergosterol acetate (**2**), and (3*R*)-3-(1*H*-indol-3-ylmethyl)-3, 4-dihydro-1*H*-1,4-benzodiazepine-2,5-dione (**3**), from the column fractions of the crude ethyl acetate extract of the culture of a marine sponge-associated fungus, *Aspergillus fischeri* MMERU 23. The structure of **4** was elucidated by the interpretation of 1D and 2D NMR spectral data and high-resolution mass spectrum. The absolute configuration of the stereogenic carbon in **3** was proposed to be the same as those of the co-occurring congeners on the basis of their biogenetic consideration and was supported by the comparison of its sign of optical rotation with those of its steroisomers. The crude ethyl acetate extract and **2** were evaluated, together with acetylaszonalenin (**5**) and helvolic acid (**6**), which were previously isolated from the same extract, for the in vivo antinociceptive activity in the mice model. The crude ethyl acetate extract exhibited antinociceptive activity in the acetic acid-induced writhing and formalin tests, while **2**, **5**, and **6** displayed the effects in the late phase of the formalin test. On the other hand, neither the crude ethyl acetate extract nor **2**, **5**, and **6** affected the motor performance of mice in both open-field and rotarod tests. Additionally, docking studies of **2**, **5**, and **6** were performed with 5-lipoxygenase (5-LOX) and phosphodiesterase (PDE) enzymes, PDE4 and PDE7, which are directly related to pain and inflammatory processes. Molecular docking showed that **6** has low affinity energy to PDE4 and PDE7 targets while retaining high affinity to 5-LOX. On the other hand, while **2** did not display any hydrogen bond interactions in any of its complexes, it achieved overall better energy values than **6** on the three antinociceptive targets. On the other hand, **5** has the best energy profile of all the docked compounds and was able to reproduce the crystallographic interactions of the 5-LOX complex.

## 1. Introduction

The fungi of the genus *Aspergillus* (Family Aspergillaceae), which are widely distributed in terrestrial and marine environments, are the most studied genus as a source of secondary metabolites. In the marine environment, *Aspergillus* species are found to be associated with many macroorganisms such as macroalgae, marine sponges, corals, mangrove, and other marine invertebrates [1]. Members of the *Aspergillus* genus are producers of a myriad of secondary metabolites, including polyketides, peptides, alkaloids, and terpenoids, many of which exhibited a variety of biological and pharmacological activities such as cytotoxicity, antibacterial, antifungal, antiviral, antibiofilm, and anti-inflammatory activities, and they can also act as enzyme inhibitors [2,3].

**Figure 1:**
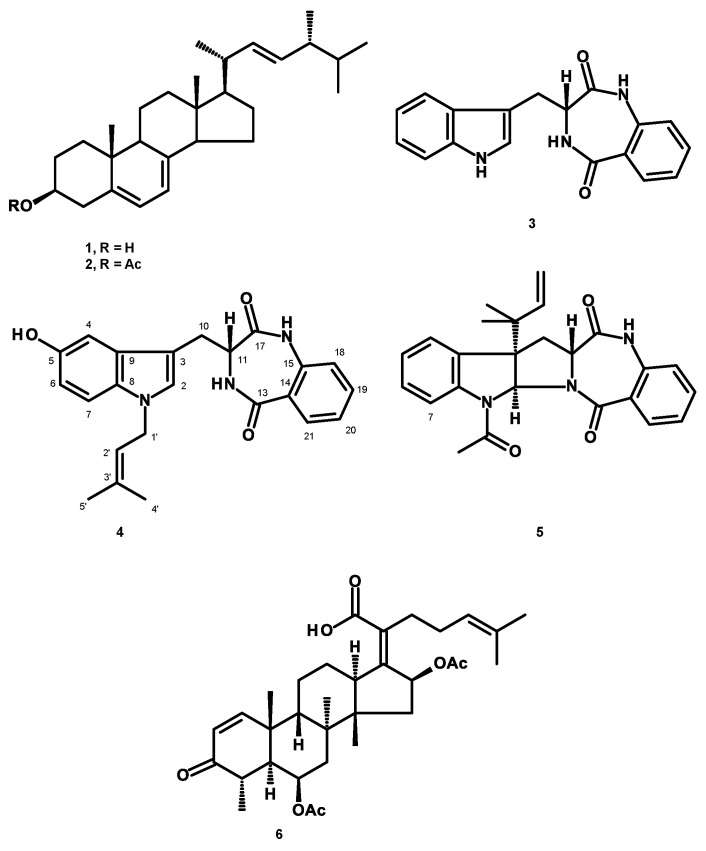
Structures of ergosterol (**1**), ergosterol acetate (**2**), (3*R*)-3-(1*H*-indol-3-ylmethyl)-3, 4-dihydro-1*H*-1,4-benzodiazepine-2,5-dione (**3**), hydroxytakakiamide (**4**), acetylasznalenin (**5**), and helvolic acid (**6**).

In our ongoing search for bioactive compounds from marine-derived fungi from the Gulf of Thailand, we have investigated secondary metabolites from the solid rice culture extract of a marine sponge-associated *Aspergillus fischeri* MMERU 23, which was isolated from the marine sponge *Hyrtios erecta*, collected from Rok Nai Island in the Andaman Sea of Krabi province, Thailand, and isolated two prenylated índole derivatives, aszonalenin and acetylaszonalenin (**5**), a meroditerpene aszonapyrone A, and helvolic acid (**6**) (Figure 1). All the isolated compounds were tested for their affinity against *Leishmania major* pteridine reductase 1 (PTR1) via thermal shift assay [4].

Re-examination of the column fractions which had not been investigated in the first study led to the isolation of ergosterol (**1**), ergosterol acetate (**2**), (3*R*)-3-(1*H*-indol-3-ylmethyl)-3, 4-dihydro-1*H*-1,4-benzodiazepine-2,5-dione (**3**), and an unreported hydroxytakakiamide (**4**) (Figure 1).

In our quest for naturally occurring antinociceptive compounds from marine resources, we have noticed that most of the research works in this field have been conducted with crude extracts from marine macroalgae [5,6,7,8], marine sponges [9], and to a lesser extent, with compounds isolated from macroalgae [10,11] and marine sponges [12]. To the best of our knowledge, there has been no report on the in vivo antinociceptive activity of crude extracts of marine-derived fungi. However, Wang et al. [13] have reported the anti-inflammatory and analgesic activities of 2-(2-hydroxypropanamido) benzoic acid, which was isolated from the fermentation broth of the marine fungus *Penicillium chrysogenum*.

Consequently, the crude ethyl acetate extract of the culture of *A. fisheri* MMERU 23 and its constituents, i.e., **2** and the previously isolated **5** and **6** (Figure 1), which were isolated in sufficient quantities for in vivo assays, were tested for their antinociceptive activity via acetic acid-induced writhing and formalin tests in a mice model, while the motor performance of mice was evaluated by the rotarod and open-field tests. The effects of **2**, **5**, and **6** on pain were further investigated by a molecular docking study. Another reason that we chose a fungal sterol **2** and a nortriterpenoid **6** as the target compounds is that both of them share the same perhydrocyclopentanophenantrene scaffold as the higher plant sterols, β-sitosterol and stigmasterol (Figure S1), which have exhibited antinociceptive activity [14,15,16].

## 2. Results and Discussion

The structures of ergosterol (**1**) (Figures S2 and S3), ergosterol acetate (**2**) (Figures S4 and S5) [17], and (3*R*)-3-(1*H*-indol-3-ylmethyl)-3, 4-dihydro-1*H*-1,4-benzodiazepine-2,5-dione (**3**) (Figures S6 and S7) [18] were elucidated by comparison of their ^1^H and ^13^C NMR spectra and other physical data with those reported in the literature.

Compound **4** was isolated as a white amorphous solid, and its molecular formula, C_23_H_23_N_3_O_3_, was established based on the (-)-HRESIMS *m/z* 338.1657 [M-H]^+^ (calculated for C_23_H_22_N_3_O_3_, 338.1661) (Figure S13), indicating 14 degrees of unsaturation. The ^1^H and ^13^C NMR spectra of **4** in CDCl_3_ (Table 1, Figures S8 and S9) resembled those of takakiamide, a 3-[1-(3-methylbut-2-enyl)indol-3-yl]-3,4-dihydro-1*H*-1,4-benzodiazepine-2, 5-dione, previously isolated from the culture extract of the algicolous fungus *Neosartorya takakii* KUFC 7898 [19], except for those of the benzene ring of the indole moiety, which indicated the presence of 1,2,4-trisubstituted instead of 1,2-disubstituted in takakiamide. That the hydroxyl group was on C-5 in **4** was evidenced by the presence of a doublet at δ_H_ 6.95 (*J* = 2.0 Hz/δ_C_ 103.1, C-4), a double doublet at δ_H_ 6.79 (*J* = 8.5, 2.0 Hz/δ_C_ 111.9, C-6), and a doublet at δ_H_ 7.16 (*J* = 8.5 Hz/δ_C_ 110.5, C-7). This was supported by COSY correlations from H-6 to H-4 and H-7 and HMBC correlations from H-4 to C-5, C-6, and C-8 (δ_C_ 131.8), H-6 to C-4 and C-8, and H-7 to C-5 (δ_C_149.7) and C-9 (δ_C_128.5). That the 3-methylbut-2-enyl group was on N-1 of the indole moiety was corroborated by the HMBC correlation from H_2_-1′ (δ_H_ 4.58, *J* = 7.0 Hz) to C-8. Therefore, the planar structure of **4** was elucidated as 3-[1-(3-methylbut-2-enyl), 5-hydroxy-indol-3-yl]-3,4-dihydro-1*H*-1,4-benzodiazepine-2, 5-dione. The absolute configuration at C-11 was proposed to be the same as that of C-11 of takakiamide on the basis of the biogenetic considerations and also on the same sign of optical rotation, i.e., levorotatory ([α]_D_^20^ -213 (*c* 0.06, CHCl_3_) for takakiamide and [α]_D_^22^ -11 (*c* 0.05, MeOH) for **4**). Moreover, Yin et al. [20], in their biosynthetic study of acetylaszonalenin (**5**) in *Neosartorya fischeri*, have identified the biosynthetic gene cluster by genomic mining. They have proven that **3** was a precursor of aszonalenin and acetylaszonalenin (**5**). Moreover, through the feeding experiment, they have found that L-Trp was converted to D-Trp before or during formation of the cyclic dipeptide (*R*)-benzodiazepinedione (**3**). They speculated that this happened during the dipeptide synthesis catalyzed by an epimerase domain of the non-ribosomal peptide synthetase AnaPS from *N. fischeri*.

Thus, the co-occurrence of **4** with **3** and **5** in *A. fisheri* MMERU 23 also supports the fact that the absolute configuration at C-11 in **4** is the same as that of C-11 in **3** and **5**, i.e., 11*R*. A literature search revealed that **4** has never been previously reported, although a similar compound named asperdiazapinone D, with a hydroxyl group on C-6 and a 11*S* configuration, has been previously reported from the mycelial extract of the soil fungus, *Aspergillus* sp. PSU-RSPG185. Interestingly, asperdiazapinone D is dextrorotatory ([α]_D_^25^ +137.0, (*c* 0.50, MeOH) [21], thus corroborating the 11*R* configuration in **4**. Therefore, **4** was named hydroxytakakiamide.

**Figure 2:**
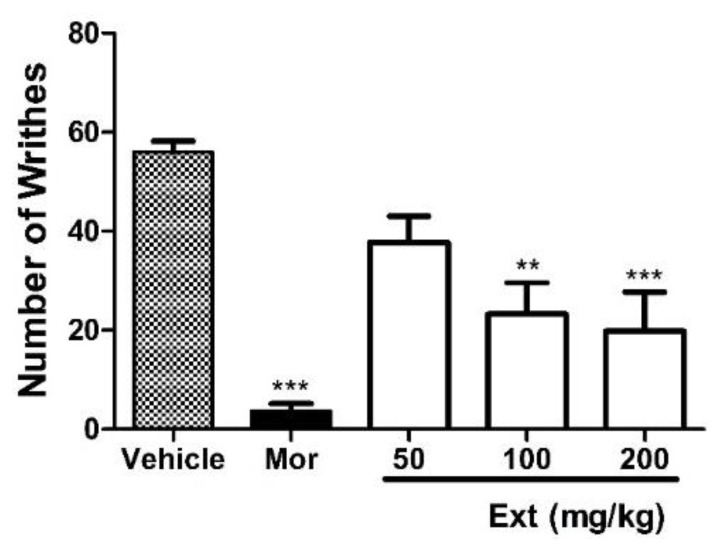
Effects of oral administration of the crude ethyl acetate extract of *A. fischeri* MMERU 23 (Ext) in acetic acid-induced writhing. Mice were divided in three groups, 6 mice per group (n = 6). The treatment group received 50, 100, and 200 mg/kg of Ext, p.o., the control group received a vehicle 60 min before intraperitoneal injection with 0.8% acetic acid (injected at time zero), and the third group received a reference drug, morphine (Mor, 5 mg/kg, s.c.), 40 min before intraperitoneal injection with 0.8% acetic acid. Data are expressed as mean ± SEM. ** Significantly different from control group (*p* < 0.05). *** Significantly different from control group (*p* < 0.0001) as determined by ANOVA and followed by Bonferroni’s test.

The antinociceptive effect of the crude ethyl acetate extract of *A. fischeri* MMERU 23 (Ext) was first evaluated by an acetic acid-induced writhing test in mice. Oral administration of Ext (50, 100, and 200 mg/kg), one hour before the injection of acetic acid, produced a significant inhibition of writhing in mice (Figure 2). Morphine, a positive control, produced a significant inhibition of the writhing response (*p* < 0.0001).

**Figure 3:**
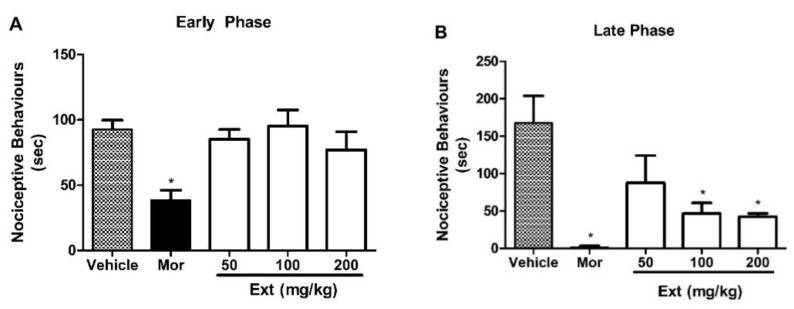
Effects of treatment with the crude extract of *A. fischeri* MMERU 23 (Ext) in the formalin test. Panels (**A**) and (**B**) represent the effects of Ext in the early and late phases of the formalin test in mice, respectively. Mice were orally administered with 50, 100, and 200 mg/kg of Ext or vehicle (control group) 60 min before a formalin injection (injected at time zero). Morphine (Mor, 5 mg/kg, s.c.) was used as a reference drug. Data are expressed as means ± S.E.M.; n = 6 mice per group. * Significantly different from the control group (*p* < 0.05), as determined by ANOVA and followed by Bonferroni´s test.

Since the abdominal writhing response can be also altered by muscle relaxants, neuroleptics, and other drugs, the antinociceptive effect was then confirmed via the formalin test to avoid the error of interpretation. Intraplantar injection of formalin to the mice induced a biphasic behavioral response, i.e., the first (early) phase (from 0 to 5 min) (Figure 3A) and the second (late) phase (from 15 to 30 min) (Figure 3B). The results showed that treatment with Ext (50, 100, and 200 mg/kg) did not exhibit any antinociception in the first (early) phase (Figure 3A). It is worth mentioning that in the first (early) phase, a direct chemical stimulation promoted by formalin occurs in the nociceptors of afferent fibers C and Aδ, which is associated with the release of excitatory amino acids, nitric oxide (NO) [22], and substance P [23]. On the contrary, oral administration of Ext resulted in antinociception (*p* ˂ 0.05) in the late phase (Figure 3B). Since several inflammatory mediators such as histamine, bradykinin, serotonin, leucotrienes, and prostaglandins, which are responsible for sensitizing and stimulating nociceptors and inducing nociceptive behavior, are released in the late phase [24,25], it is concluded that the antinociceptive effect of Ext in the late phase was due to the inhibition of the proinflammatory mediators. On the other hand, the reference compound, morphine, exhibited antinociceptive effects in both phases.

Compound **2**, together with **5** and **6**, which were previously isolated from the culture extract of the same fungus **[4]**, were tested for antinociceptive activity since they were isolated in sufficient quantities for the in vivo tests. However, due to the limited quantity of **2**, **5**, and **6** and the fact that, for pain study, the formalin test is more specific than the acetic acid-induced writhing test, they were subject to only the formalin test.

**Figure 4:**
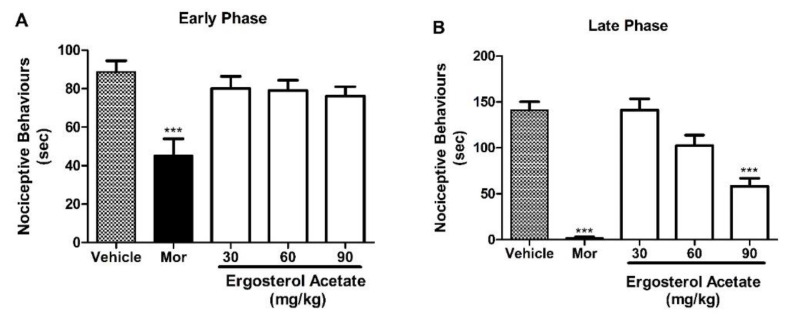
Effects of treatment with **2** in the formalin test. Panels (**A**) and (**B**) represent the effects of **2** on the early and late phases of the formalin test in mice, respectively. Mice were treated with **2** (30, 60, and 90 mg/kg) or vehicle (control group) via the oral route 60 min before a formalin injection (injected at time zero). Morphine (Mor, 5 mg/kg) was used as a reference drug. Data are expressed as means ± S.E.M.; n = 6 mice per group. *** Significantly different from the control group (*p* < 0.0001) as determined by ANOVA and followed by Bonferroni´s test.

**Figure 5:**
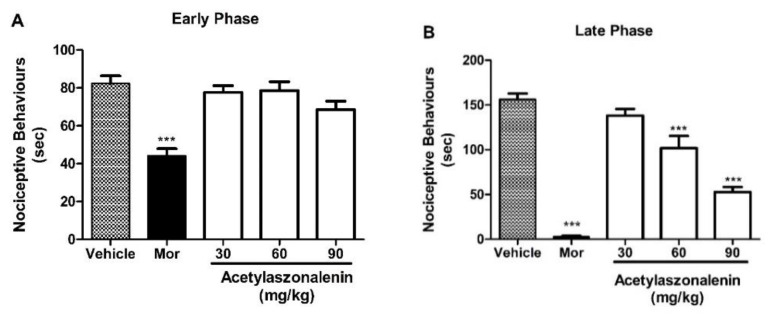
Effects of treatment with **5** in the formalin test. Panels (**A**) and (**B**) represent effects of **5** on the early and late phases of the formalin test in mice, respectively. Mice were treated with **5** (30, 60, and 90 mg/kg) or vehicle (control group) via the oral route 60 min before a formalin injection (injected at time zero). Morphine (Mor, 5 mg/kg) was used as a reference drug. Data are expressed as means ± S.E.M.; n = 6 mice per group. *** Significantly different from the control group (*p* < 0.0001) as determined by ANOVA and followed by Bonferroni´s test.

**Figure 6:**
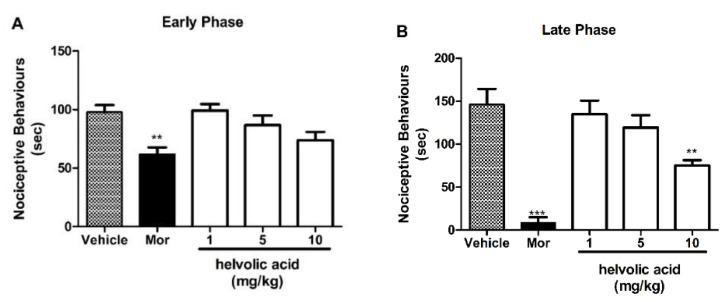
Effects of treatment with **6** in the formalin test. Panels (**A**) and (**B**) represent the effects of **6** on the early and late phases of the formalin test in mice, respectively. Mice were orally administered with **6** (1, 5, and 10 mg/kg) or vehicle (control group) before a formalin injection. Morphine (Mor, 5 mg/kg, s.c.) was used as a reference drug. Data are expressed as means ± S.E.M.; n = 6 mice per group. ** Significantly different from the control group (*p* < 0.05). *** Significantly different from the control group (*p* < 0.0001) as determined by ANOVA followed by Bonferroni´s test.

Oral administration of **2** (90 mg/kg, p.o.) (Figure 4), **5** (60 and 90 mg/kg, p.o.) (Figure 5), and **6** (90 mg/kg, p.o.) (Figure 6) did not show any effect in the early phase (A); however, a significant antinociceptive effect in the late phase (B) of the formalin test was observed, suggesting a direct involvement of these compounds in the inhibition of proinflammatory mediators. The positive control, morphine (5 mg/kg, s.c.), produced an antinociception in both early and late phases.

**Figure 7:**
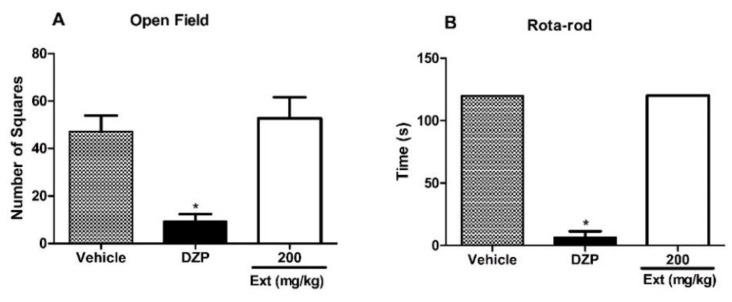
Effects of Ext in the open field (**A**) and rotarod (**B**) tests. Mice were orally administered with 200 mg/kg of Ext or vehicle (control group) 60 min before the evaluation. Diazepam (DZP, 10 mg/kg) was used as a reference drug. Data are expressed as means ± S.E.M.; n = 6 mice per group. * Significantly different from the control group (*p* < 0.05), as determined by ANOVA and followed by Bonferroni’s test.

In order to rule out that any motor or neurological impairments were responsible for the lack of response caused by Ext in the nociceptive tests, the effects of Ext on the motor function were evaluated via open-field and rotarod tests. The open-field test is used to study the neurobiological basis of anxiety, as well as for screening novel drug targets and anxiolytic compounds [26]. On the other hand, the rotarod test is used to assess motor coordination and balance in rodents, which provides a quick and simple estimation of neuromuscular coordination [27]. The results showed that oral administration of 200 mg/kg of Ext did not affect the motor performance of mice in both open-field and rotarod tests (Figure 7).

**Figure 8:**
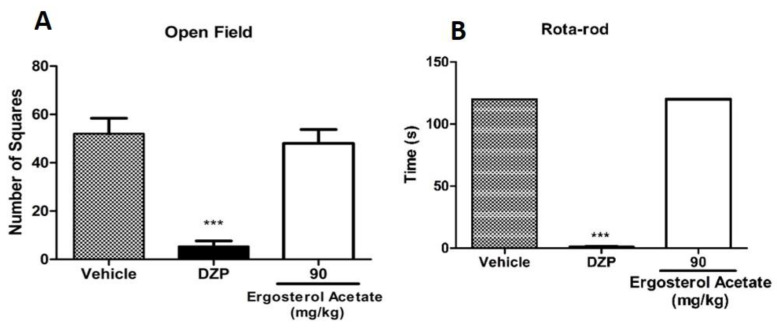
Effects of **2** in the open field (**A**) and rotarod (**B**) tests. Mice were treated with **2** (90 mg/kg) or vehicle (control group) by oral route 60 min before the evaluation. Diazepam (DZP; 10 mg/kg) was used as a reference drug. Data are expressed as means ± S.E.M.; n = 6 mice per group. *** Significantly different from the control group (*p* < 0.0001) as determined by ANOVA and followed by Bonferroni´s test.

**Figure 9:**
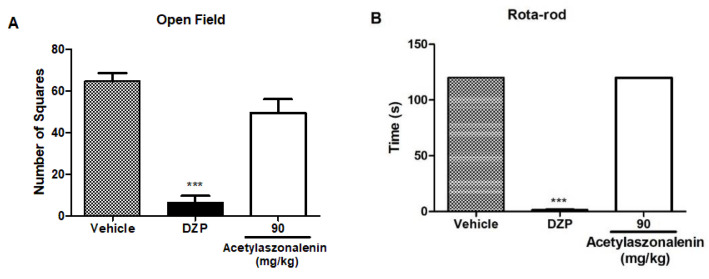
Effect of **5** in the open-field (**A**) and rota rod (**B**) tests. Mice were treated with **5** (90 mg/kg) or vehicle (control group) by oral route 60 min before the evaluation. Diazepam (DZP, 10 mg/kg) was used as a reference drug. Data are expressed as means ± S.E.M.; n = 6 mice per group. *** Significantly different from the control group (*p* < 0.0001) as determined by ANOVA and followed by Bonferroni´s test.

**Figure 10:**
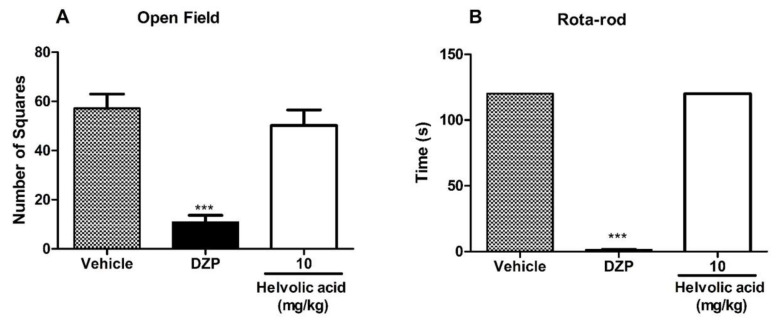
Effect of **6** in the open field (**A**) and rotarod (**B**) tests. Mice were treated with **6** (10 mg/kg) or vehicle (control group) by oral route 60 min before the evaluation. Diazepam (DZP, 10 mg/kg) was used as a reference drug. Data are expressed as means ± S.E.M.; n = 6 mice per group. *** Significantly different from the control group (*p* < 0.0001) as determined by ANOVA and followed by Bonferroni´s test.

Compounds **2** (90 mg/kg), **5** (90 mg/kg) and **6** (10 mg/kg) were also evaluated to verify if they affected motor or neurological impairments by open field and rotarod tests. The results showed that neither of them affected the motor performance of mice in both open field and rotarod tests (Figure 8, Figure 9 and Figure 10).

Since **2**, **5**, and **6** displayed antinociceptive activity in the late phase of the formalin test, these compounds must have a specific inhibition of proinflammatory mediators, suggesting that they are responsible for the antinociceptive effects of the crude acetyl acetate extract of *A. fischeri* MMERU 23 in the mice model.

The mechanisms related to pain and inflammation have been widely discussed in the literature on drugs discovery studies [28,29,30], and they are generally associated with their role in the metabolic route of inflammation via key enzymes such as lipoxygenase, which produces effectors of pain and inflammation in osteoarthritis as well as many other diseases since they are involved in the metabolism of arachidonic acid to inflammatory fatty acids, e.g., 5-hydroperoxyeicosatetraenoic acid (5-HPETE) leading to leukotriene signaling [31], while the phosphodiesterase enzymes (PDE) metabolize the intracellular inflammatory second messengers cyclic adenosine monophosphate (cAMP) and cyclic guanosine monophosphate (cGMP), with PDE4 specifically hydrolyzing the 3′, 5′-phosphodiester bond of cAMP to yield 5′-AMP. On the other hand, PDF7 is responsible for the final inactive metabolites of the cAMP/cGMP cycle (5′-AMP and 5′-GMP). Although the inhibition of PDF7 is unable to influence proinflammatory cells, it boosts the inhibitory effect of other cAMP-elevating drugs [32,33]. Since some phytosterols [34] and nor-triterpenenes [35] have been found to exhibit anti-inflammatory activity, computational methods can be used to hypothesize the intermolecular interactions of **2** and **6** towards these targets and can therefore help to identify promising anti-inflammatory inhibitors.

**Figure 11:**
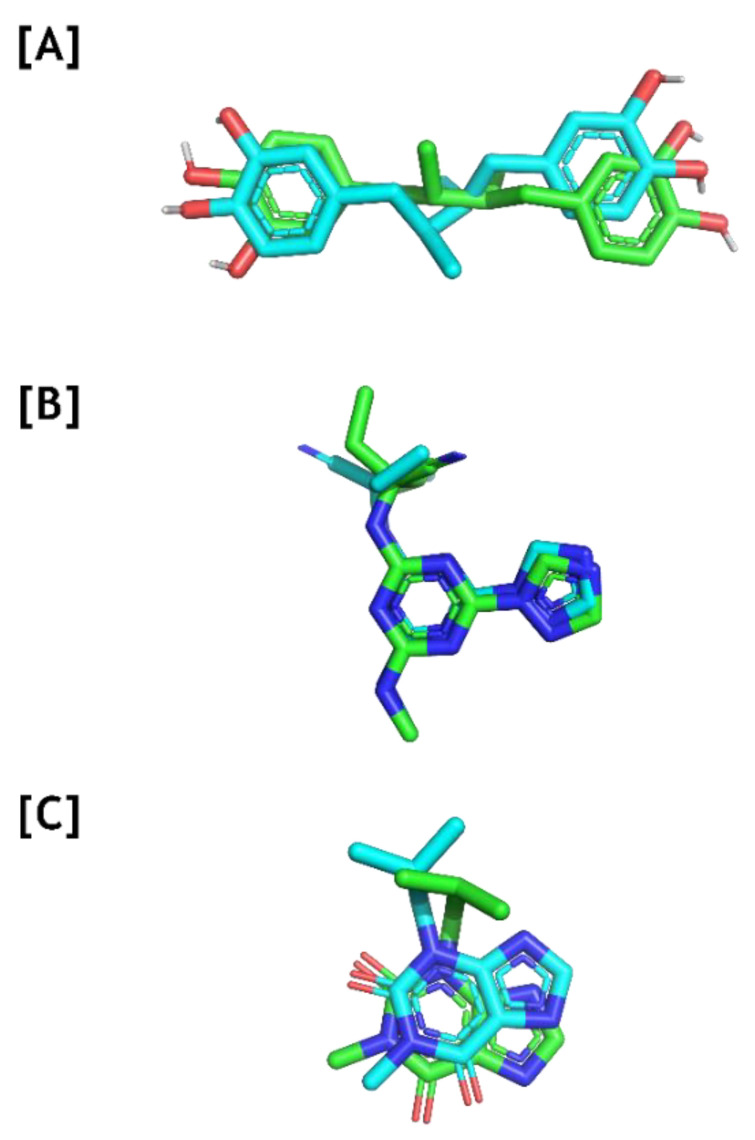
Re-docking of the ligands of 5-LOX [**A**] (PDB ID: 6N2W; RMSD = 1.79 Å), PDE4 [**B**] (PDB ID: 4KP6; RMSD = 1.95 Å), and PDE7 [**C**] (PDB ID: 1ZKL; RMSD = 0.94 Å) on Autodock Vina software. The crystallographic ligand poses are shown by green sticks while the re-docked poses are shown by blue sticks.

In order to further evaluate the antinociceptive activity of **2**, **5**, and **6**, docking studies were performed with three enzymes directly related to pain and inflammatory processes, *viz*. 5-lipoxygenase (5-LOX), PDE4, and PDE7 [36,37,38]. As the 3D structures of 5-LOX, PDE4, and PDE7 contain crystallographic ligands, a re-docking was performed to evaluate the efficacy of Autodock Vina search parameters so that they can reproduce the experimental interaction poses. Root means square deviation (RMSD) values of less than 2 Å, which are commonly used to consider a ligand pose similar to the native state [39], were used in this study. The RMSD and energy values obtained for the complexes are shown in Table 2 and Figure 11.

**Figure 12:**
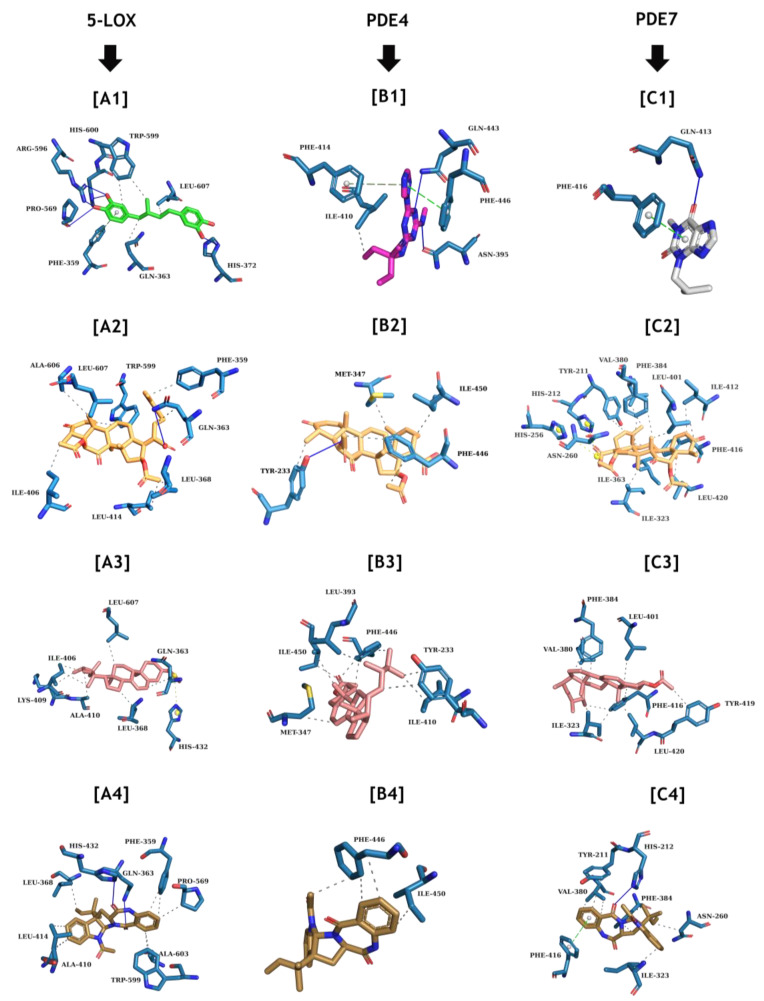
Comparison of the interaction patterns for the crystallographic ligands versus **2**, **5**, and **6** on their active sites. [**A1**] 5-LOX ligand (PDB: 6N2W) on green sticks, [**B1**] PDE4 ligand (PDB: 4KP6) on violet sticks, and [**C1**] PDE7 ligand (PDB: 1ZKL) on grey sticks. The compounds are paired with the most favorable pose of **6** (orange-colored sticks), **2** (salmon-colored sticks), and **5** (olive green-colored sticks) and on each target as follows: [**A2**] **6** on 5-LOX (−8.1 kcal/mol), [**B2**] **6** on PDE4 (−3.3 kcal/mol), and [**C2**] **6** on PDE7 (−3.0 kcal/mol); [**A3**] **2** on 5-LOX (−8.7 kcal/mol), [**B3**] **2** on PDE4 (−5.5 kcal/mol), and [**C3**] **2** on PDE7 (−6.8 kcal/mol); [**A4**] **5** on 5-LOX (−9.0 kcal/mol), [**B4**] **5** on PDE4 (−7.8 kcal/mol), and [**C4**] **5** on PDE7 (−8.6 kcal/mol). Protein residues are shown in blue; hydrogen bonds are shown in navy blue lines and hydrophobic interactions are shown in black dashed lines.

The re-docking results showed that Autodock Vina software was capable of achieving docking poses that are spatially close to the experimental conformations. Therefore, the same method was used to dock **2**, **5**, and **6** on the targets’ active sites and also evaluated those interactions. Figure 12 shows the interaction patterns found in the most favorable poses for **2**, **5**, and **6** as well as on 5-LOX, PDE4, and PDE7.

Compound **6** was able to maintain its hydrophobic interactions with PHE-359, GLN-363, and TRP-599 residues and a hydrogen bond with a LEU-607 residue, similar to the interaction of 5-LOX with masoprocol (a potent inhibitor) described in the crystallographic structure used in this work (PDB: 6N2W), while also establishing a new hydrogen bond with the GLN-363 residue, which is not visible in the experimental structure.

The most favorable pose for **6** at the 5-LOX binding site had an energy score of −8.1 kcal/mol on Autodock Vina. At the PDE4 binding site, **6** only maintains an interaction with PHE-446 residue, which is in common with its crystallographic ligand (a potent and selective inhibitor), while establishing a hydrogen bond with TYR-223, an interaction that does not happen in the crystal structure. Although there are no pi-stacking interactions, it was able to interact with MET-347, ILE-450, and PHE-446 residues, with an overall energy score of -3.3 kcal/mol on Autodock Vina.

When compared to the non-selective inhibitor, 3-isobutyl-1-methylxanthine, in its crystal structure of the complex with PDE7, **6** is not only able to maintain an interaction with PHE-416 but also forms more 12 hydrophobic interactions with TYR-221, HIS-212, HIS-256, ASN-260, ILE-323, VAL-380, PHE-384, LEU-401, ILE-412, and LEU-420. The most favorable complex of **6** and PDE7 scored a value of −3.3 kcal/mol on Autodock Vina.

Molecular docking showed that **6** has low affinity energy to PDE4 and PDE7 targets while retaining high affinity to 5-LOX as it was able to reproduce some of the interaction patterns as observed in the crystallographic structures available and scored good energy results. Interestingly, there are reports suggesting that the dual inhibition of PDE4 and PDE7 is a potential therapeutic strategy for decreasing neuroinflammation [40].

When compared with the results obtained with **6**, the docking routines with **2** showed that while this compound did not display any hydrogen bond interactions in any of its complexes, it achieved overall better energy values on the three antinociceptive targets. Due to the lipophilic property of **2**, together with the presence of a conjugated 1, 4-cyclohexadiene moiety, this compound mainly interacts with catalytic hydrophobic residues such as LEU-607 on 5-LOX, PHE-446 on PDE4, and PHE-416 on PDE7, while maintaining interactions with other nearby hydrophobic residues. Such interactions are commonly observed for compounds that interact and modulate lipophilic pockets in proteins. The impact of these sole interactions on nociceptive activity is still unknown; however fungal sterols are known for their antioxidant properties, thus supporting the results obtained from the nociceptive tests.

On the other hand, **5** has the best energy profile among all the docked compounds. Compound **5** was able to reproduce the crystallographic interactions of the 5-LOX complex with PHE-359, GLN-363, PRO-569, and TRP-599, while also showing extra interactions with LEU-368, ALA-410, LEU-414, HIS-432, and ALA-603. Compound **5** was also able to reproduce the interaction with PHE-446 from the PDE4 complex, while also establishing a hydrophobic interaction with ILE-450 of this protein. Moreover, when compared to the PDE7 complex, **5** was able to maintain the PHE-416 catalytic interaction, while showing a wider array of interactions, including TYR-211, HIS-212, ASN-260, ILE-323, VAL-380, and PHE-384.

Integration of the in vitro/in vivo with in silico studies has showed good results in the identification of promising anti-inflammatory and antinociceptive compounds [41]. Thus, the data generated from this study indicated that **2**, **6**, and especially **5** have potential in the development of antinociceptive and anti-inflammatory agents.

## 3. Experimental Section

### 3.1. General Procedure

Optical rotations were measured on Digital polarimeter P-2000-Na (ABL and A-JASCO, Kraków, Poland). ^1^H and ^13^C NMR spectra were recorded at ambient temperature on a Bruker AMC instrument (Bruker Biosciences Corporation, Billerica, MA, USA) operating at 300 and 75 MHz, respectively, and on a Bruker Avance III spectrometer (Bruker Biosciences Corporation, Billerica, MA, USA) operating at 500 and 125 MHz, respectively. Chemical shifts were referenced to the residual peaks of the deuterated solvents. High-resolution mass spectra were measured with a micrOTOF (Bruker Daltonics, Bremen, Germany). A Merck (Darmstadt, Germany) silica gel GF_254_ was used for preparative TLC, and a Merck Si gel 60 (0.2–0.5 mm) was used for column chromatography. LiChroprep silica gel and Sephadex LH-20 were used for column chromatography.

### 3.2. Isolation of the Compounds

The isolation and identification of *A. fischeri* MMERU 23, as well as the fractionation of the ethyl acetate extract of the culture of *A. fischeri* MMERU 23, were previously described by Cardona et al. [4]. The fractions from the first column chromatography of the culture extract of *A. fischeri* MMERU 23 that had not been examined in our previous work [4] were re-examined as follows: Fraction (Fr) 1 (2.44 g) was precipitated in a mixture of petrol and CHCl_3_ to give 200 mg of white solid, which was purified via TLC (silica gel G_254_, CHCl_3_-Me_2_CO-HCO_2_H, 8:2:0.1) to give 19.0 mg of ergosterol (**1**) and 59.0 mg of ergosterol acetate (**2**). Fr. 21 (1.60 g) was applied over a Sephadex LH-20 column (15 g) and eluted with MeOH, wherein 32 subfractions (Sfrs) of 100 mL were collected. Sfr. 22 (160.0 mg) was purified by TLC (silica gel G_254_, CHCl_3_-Me_2_CO-HCO_2_H, 8:2:0.1) to give 5.0 mg of **3** and 7.0 mg of **4**.

#### Hydroxytakakiamide (**4**)

White amorphous solid [α]^22^_D_ -11 (*c* 0.05, MeOH) for ^1^H and ^13^C NMR (see Table 1); (-)-HRESIMS *m/z* 388.1657 [M-H]^−^ (calculated for C_23_H_22_N_3_O_3_, 388.1661).

### 3.3. Antinociceptive Activity

#### 3.3.1. Drugs and Reagents

Morphine sulphate was purchased from Cristália^®^ (São Paulo, Brazil). Diazepam was purchased from União Química^®^ (São Paulo, Brazil), formaldehyde from Synth^®^ (São Paulo, Brazil), and saline (0.9% physiological sodium chloride solution) from Farmace^®^ (Ceará, Brazil). A solution of 2.5% formalin was prepared with formaldehyde in saline. The extract was dissolved in DMSO and diluted with saline to give 5% *v/v* of DMSO concentration.

#### 3.3.2. Animals

The experiments were carried out using male Swiss mice (two months old) with the weight of 25–35 g, which were obtained from the State University of Feira de Santana, Brazil. The mice were placed in appropriate boxes and maintained at 22 ± 2 °C under a 12 h light–dark cycle with free access to food and water ad libitum until use. All experiments were carried out according to the protocol of the International Association for the Study of Pain (IASP) for the use of animals in the investigation of pain [42] after receiving approval by the Ethics Committee (006%2013) of the State University of Feira de Santana.

#### 3.3.3. Drug Administration

Ext and vehicle (saline + 1% *v/v* Tween 80) were orally administered one hour before the experiment. Reference drugs (morphine and diazepam) were dissolved in saline and administered (s.c.) 20 min after administration of Ext or vehicle.

#### 3.3.4. Acetic Acid-Induced Writhing Test

The acetic acid-induced writhing test was performed following the previously described method [43] with modification. Briefly, mice were orally administered with Ext (50, 100, and 200 mg/kg) or vehicle (control group) or morphine (5 mg/kg, s. c.). One hour after the Ext treatment, mice were administered (i. p.) with acetic acid (0.8% *v/v* in saline, 10 mL/kg) and placed individually in a 24 cm-diameter chamber for observation. The intensity of nociceptive behavior was quantified by counting the total numbers of writhes (abdominal contortions) during 30 min after administration of the nociceptive stimulus. Abdominal contortions were characterized as contractions of the abdominal muscles associated with elongation of the body and extension of the hind limbs [44]. The antinociceptive activity was expressed as the writhing scores over 30 min.

#### 3.3.5. Formalin Test

The formalin test was performed following the previously described method [43] with modification. Briefly, mice were injected in the right hind leg with 20 µL of 2.5% formalin (0.92% formaldehyde in saline) 60 min after treatment with Ext (50, 100, and 200 mg/kg, p.o.), **2** (30, 60, and 90 mg/kg, p.o.), **5** (30, 60, and 90 mg/kg, p.o.), **6** (1, 5, and 10 mg/kg, p.o.), vehicle (control group), or morphine (5 mg/kg, s. c.). After formalin administration, mice were placed in the observation chamber. The mice were observed from 0 to 5 min (early phase) and from 15–30 min (late phase). The intensity of nociception was determined by counting the time the mouse spent licking or biting the injected paw during the observation periods [24,45].

#### 3.3.6. Open-Field Test

The open-field test was performed following the method described by Rodrigues et al. [46] with modification. In the open-field test, one hour before the observation, mice were orally administered with Ext (200 mg/kg), **2** (90 mg/kg), **5** (90 mg/kg), **6** (10 mg/kg, p.o.), vehicle, or diazepam (10 mg/kg, s.c.). The mice were placed individually in the apparatus consisting of a wooden box of 40 × 60 × 50 cm, with the floor divided into 12 identical squares. The number of squares crossed with the four paws was measured for three min.

#### 3.3.7. Rotarod Test

The rotarod test was performed according to the method previously described by Santos et al. [7] with modification. Mice were subject to a pre-selection process to choose only the mice that remained on the rotarod apparatus (consists of a balance bar) for two min at a constant speed of 16 rpm. For the rotarod assay, the selected mice received Ext (200 mg/kg, p.o.), **2** (90 mg/kg, p.o.), **5** (90 mg/kg. p.o.), **6** (10 mg/kg, p.o.), vehicle (p.o.), or diazepam (10 mg/kg, s.c.) 40 min before the test. The coordination capacity of the mice was evaluated by recording the time spent on the apparatus with a cut-off time of two min.

#### 3.3.8. Statistical Analysis

The results were presented as means ± SEM for 6 mice per group. The experimental groups were compared via a one-way ANOVA test, followed by Bonferroni’s test. The differences are considered statistically significant for values with *p* < 0.05. All data were analyzed using the GraphPad Prism^®^ software version 5.01 (GraphPad Software Inc., La Jolla, CA, USA).

### 3.4. Molecular Docking Routines

3D structures of 5-LOX (PDB ID:6N2W) [47], PDE4 (PDB ID: 4KP6) [48], and PDE7 (PDB ID: 1ZKL) [49] were obtained from the Protein Bank database [50], then submitted to molecular docking on Autodock Vina 1.2.0 [51]. The docking efficiency to predict the ligand poses for each protein complex was evaluated by the re-docking of crystallographic ligands and the superimposition of the top-ranked pose based on their RMSD values. Subsequently, **2**, **5**, and **6** were drawn, and their 3D features were built on the MarvinSketch (version Europium 7, 2020, Chemaxon, Budapest, Hungary, http://www.chemaxon.com/marvin/ accessed on 15 December 2023). The docking grid box was centered on crystallographic ligands, with a spacing grid of 1 Å. The interaction of the protein–ligand complexes was analyzed through the protein–ligand interaction profiler (PLIP) web server [52].

## 4. Conclusions

Curiously, crude extracts of cultures of marine-derived fungi and their secondary metabolites have never been evaluated for in vivo antinociceptive activity in a mouse model. The isolation of ergosterol (**1**), ergosterol acetate (**2**), and helvolic acid (**6**) from the crude ethyl acetate extract of a marine sponge-associated *A. fischeri* MMERU 23 has challenged us to investigate whether this fungal extract also exhibited in vivo antinociceptive activity, since all of the three compounds possess the same perhydrocyclopentanophenantrene scaffold as the phytoterols, β-sitosterol and stigmasterol, which exhibited antinociceptive activity. Since the crude extract of *A. fischeri* MMERU 23 exhibited significant antinociceptive activity in mice via the acetic acid-induced writhing and formalin tests, the compounds that were isolated in limited but sufficient quantity for the in vivo assay, i.e., ergosterol acetate (**2**), acetylaszonalenin (**5**), and helvolic acid (**6**), were subject to the formalin test since, for pain study, this test is more specific than the acetic acid-induced writhing test. Interestingly, not only **2** and **6**, which possess the perhydrocyclopentanophenantrene scaffold, but also the prenylated indole derivative **5** exhibited a significant antinociceptive effect in the formalin test.

Molecular docking of **2**, **5**, and **6** with 5-LOX, PDE4, and PDE7, using the Autodock Vina software, was able to identify different interactions of **2**, **5**, and **6** with different amino acid residues in the nociception targets. Although **2** and **6** share the same perhydrocyclopentanophenantrene scaffold, different functional groups, substituents, and lipophilicity in both compounds result in different interactions with the nociception targets. Interestingly, **5**, with a pentacyclic scaffold originated from a fusion of 3-methylindole and 3,4-dihydro-1*H*-1,4-benzodiazepine-2,5-dione moieties, showed better interaction patterns and energy profiles than **2** and **6**. Therefore, in silico studies of the bioactive compounds are useful tools with which to hypothesize the mechanisms underlying in vivo pharmacological activity.

## Figures and Tables

**Table 1:** ^1^H and ^13^C NMR (CDCl_3_, 500 MHz, and 125 MHz) and HMBC assignment for **4**.

Position	δ_C_, Type	δ_H_ (*J* in Hz)	COSY	HMBC
2	125.5, CH	7.01, s		C-3, 8, 9
3	107.2, C	-		
4	103.1, CH	6.95, d (2.0)	H-6	C-5, 6, 8
5	149.7, C	-		
6	111.9, CH	6.79, dd (8.5, 2.0)	H-4, 7	C-4, 8
7	110.5, CH	7.16, d (8.5)	H-6	C-5, 9
8	131.8, C	-		
9	128.5, C	-		
10a b	24.1, CH_2_	3.14, dd (6.0, 15.5) 3.39, dd (8.0, 15.5)	H-10b, 11 H-10a, 11	C-2, 3, 11, 17
11	52.7, CH	4.08, dt (6.0, 8.0)	H-10a, 10b	C-3, 13
13	168.9, CO	-		
14	125.4, C	-		
15	135.7, C	-		
17	172.0, C	-		
18	120.9, CH	6.99, d (8.0)	H-19	C-14, 20
19	133.2, CH	7.49, dd (8.0, 8.0)	H-18, 20	C-15, 21
20	125.2, CH	7.26, dd (8.0, 8.0)	H-19, 21	C-14, 18
21	131.1, CH	7.93, d (8.0)	H-20	C-13, 15, 19
1′	44.3, CH_2_	4.58, d (7.0)	H-2′	C-2, 2′, 3′, 8
2′	119.9, CH	5.33, t (7.0)	H-1′	C-4′, 5′
3′	136.1, C	-	-	
4′	25.6, CH_3_	1.74, s	-	C-2′, 3′, 5′
5′	18.0, CH_3_	1.79, s	-	C-2′, 3′, 4′
NH-16	-	8.32, s	-	C-14, 15, 17

**Table 2:** RMSD and energy values obtained through re-docking on Autodock Vina software.

Receptor	RMSD (Å)	Energy (kcal/mol)
5-LOX (PDB ID: 6N2W)	1.79	−6.8
PDE 4 (PDB ID: 4K6P	1.95	−6.7
PDE7 (PDB: ID 1ZKL)	0.94	−5.8

## Data Availability

Data are contained within the article and Supplementary Materials.

## Supplementary Materials

The following are available online at https://www.mdpi.com/article/10.3390/md22030097/s1. Figure S1: Structure of β-sitosterol and stigmasterol; Figure S2: ^1^H NMR spectrum of ergosterol (**1**); Figure S3: ^13^C NMR spectrum of ergosterol (**1**); Figure S4: ^1^H NMR spectrum of ergosterol acetate (**2**); Figure S5: ^13^C NMR spectrum of ergosterol acetate (**2**); Figure S6: ^1^H NMR spectrum of (3*R*)-3-(1*H*-indol-3-ylmethyl)-3, 4-dihydro-1*H*-1,4-benzodiazepine-2,5-dione (**3**); Figure S7: ^13^C NMR of (3*R*)-3-(1*H*-indol-3-ylmethyl)-3, 4-dihydro-1*H*-1,4-benzodiazepine-2,5-dione (**3**); Figure S8: ^1^H NMR spectrum of hydroxytakakiamide (**4**); Figure S9: ^13^C NMR spectrum of hydroxytakakiamide (**4**); Figure S10: COSY spectrum of hydroxytakakiamide (**4**); Figure S11: HSQC spectrum of hydroxytakakiamide (**4**); Figure S12: HMBC spectrum of hydroxytakakiamide (**4**); Figure S13: (-)-HRMS spectrum of hydroxytakakiamide (**4**); Figure S14: ^1^H NMR spectrum of acetylaszonalenin (**5**); Figure S15: ^13^C NMR spectrum of acetylaszonalenin (**5**); Figure S16: ^1^H NMR spectrum of helvolic acid (**6**); Figure S17: ^13^C NMR spectrum of helvolic acid (**6**).
