# Supplementary material for: Hydroxytakakiamide and Other Constituents from a Marine Sponge-Associated Fungus Aspergillus fischeri MMERU23, and Antinociceptive Activity of Ergosterol Acetate, Acetylaszonalenin and Helvolic Acid
Source: Mar Drugs. 2024 Feb 20;22(3):97. doi: 10.3390/md22030097 (PMC10971494; doi:10.3390/md22030097)

Supplementary Materials

Hydroxytakakiamide and other Constituents from a Marine Sponge-Associated Fungus *Aspergillus fischeri* MMERU23, and Antinociceptive Activity of Ergosterol Acetate, Aszonalenin and Helvolic Acid

Harol Ricardo Arias Cardona[1], Bruno Cerqueira da Silva[1], Flávia Oliveira de Lima[1], Franco Henrique Andrade Leite[1], Bruno Cruz de Souza[1], Hugo Neves Brandão[1], Jorge Maurício David[2], Clayton Queiroz Alves[3]* and Anake Kijjoa[4]*

[1] Departamento de Saúde, Universidade Estadual Feira de Santana, Av. Transnordestina, 3400-Papagaio, Feira de Santana, Bahia, Brazil; hrariasc@unal.edu.co (H.C.); farma.bruno@yahoo.com.br (B. Si.); flavia_lima2000@yahoo.com.br (F. Li.); fhaleite@uefs.br (F. Le.); bcfarma@gmail.com (B. So.); hugo@uefs.br (H. B.)

[2] Instituto de Química, Universidade Federal da Bahia, Av. Milton Santos, s/nº-Ondina, Salvador, Bahia, 40170-110, Brazil; jmdavid@ufba.br (J. D.)

[3] Departamento de Ciências Exatas, Universidade Estadual Feira de Santana, Av. Transnordestina, 3400-Papagaio, Feira de Santana, Bahia, Brazil

[4] ICBAS-Instituto de Ciências Biomédicas Abel Salazar, Universidade do Porto, Rua de Jorge Viterbo Ferreira, 228, 4050-313 Porto, Portugal

*Correspondence: ankijjoa@icbas.up.pt (A.K.); Tel. +351-962712474; cqalves@uefs.br (C. A.); Tel. +55-71-91334941

Figure S1. Structure of β-sitoterol and stigmasterol.

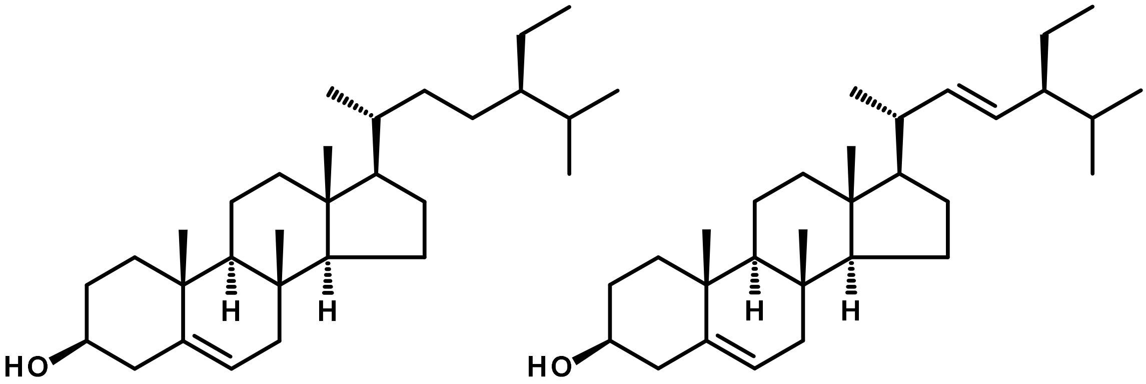

β-sitosterol stigmasterol

Figure S2. ^{1}H NMR spectrum of ergosterol (**1**) ($CDCl_3$, 300 MHz).

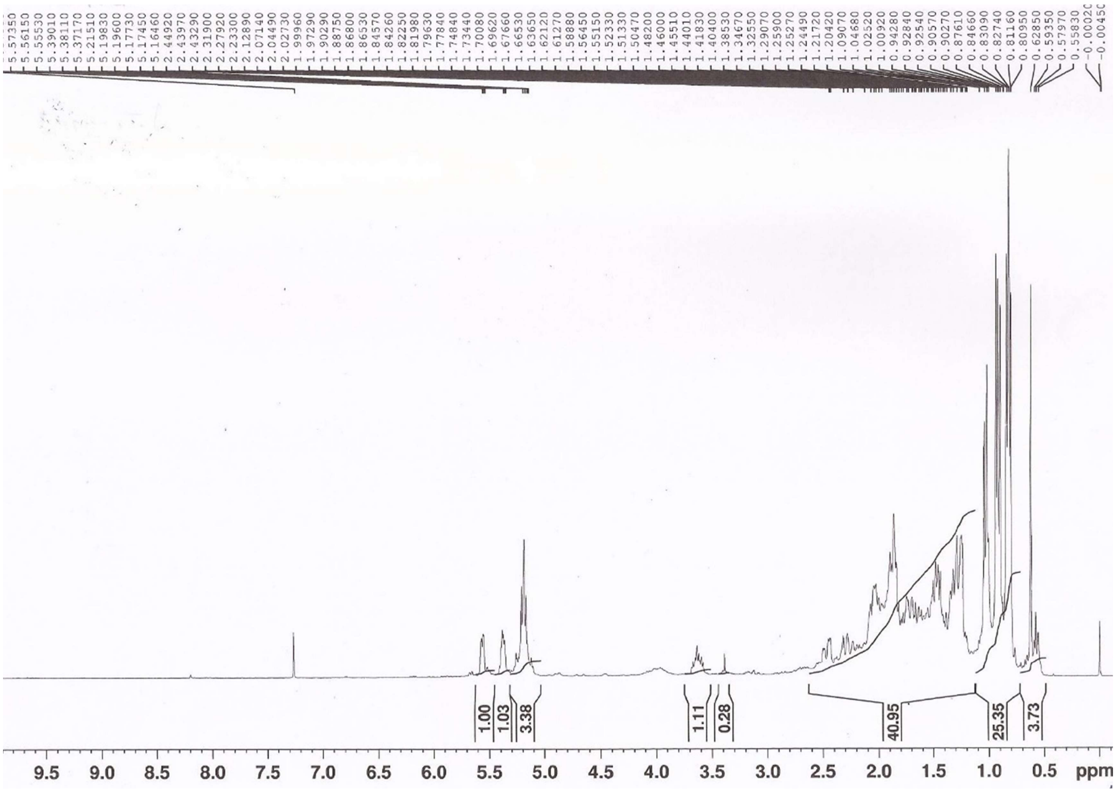

Figure S3. ^{13}C NMR spectrum of ergosterol (**1**) ($CDCl_3$, 300 MHz).

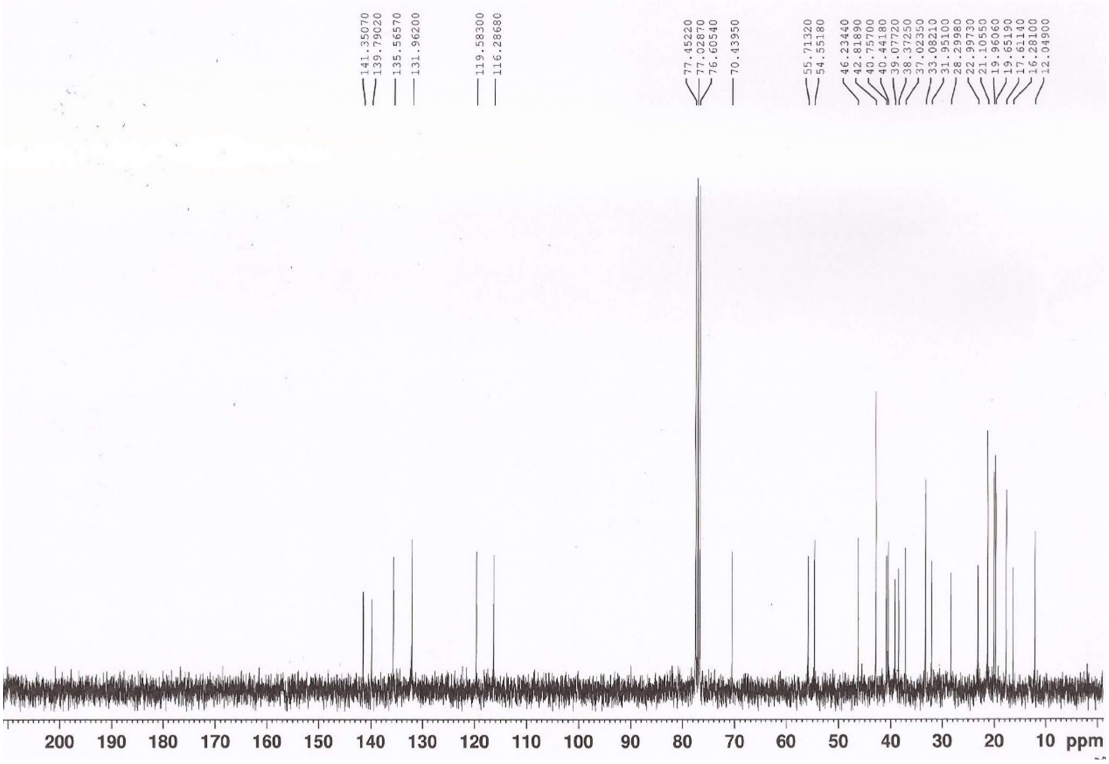

Figure S4. ^{1}H NMR spectrum of ergosterol acetate (**2**) ($CDCl_3$, 300 MHz).

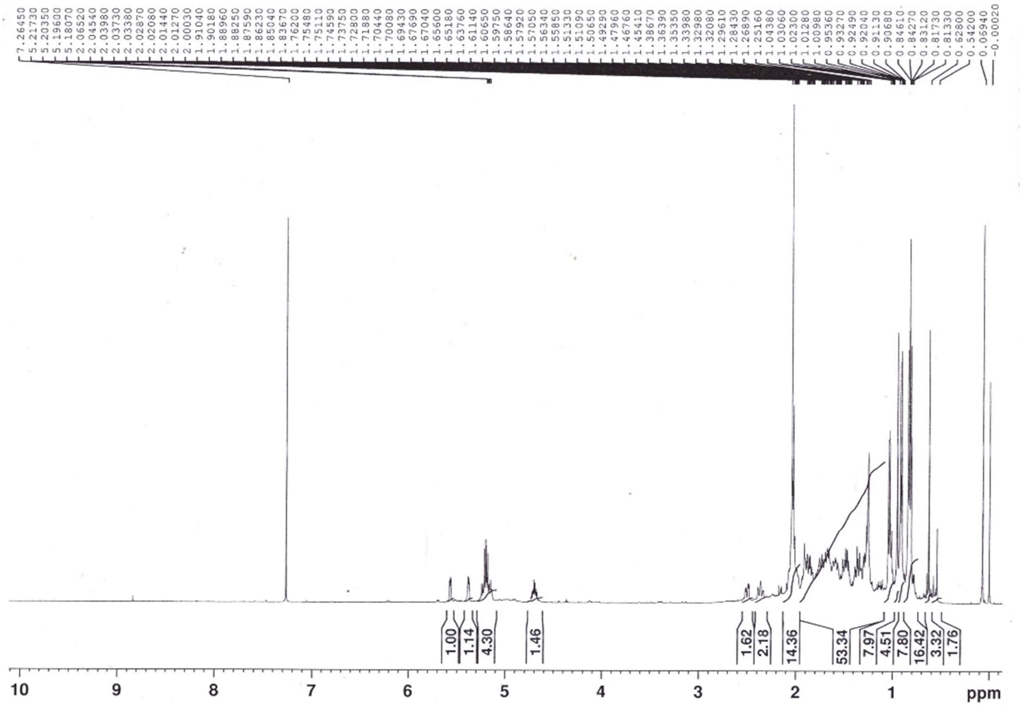

Figure S5. ^{13}C NMR spectrum of ergosterol acetate (**2**) ($CDCl_3$, 300 MHz).

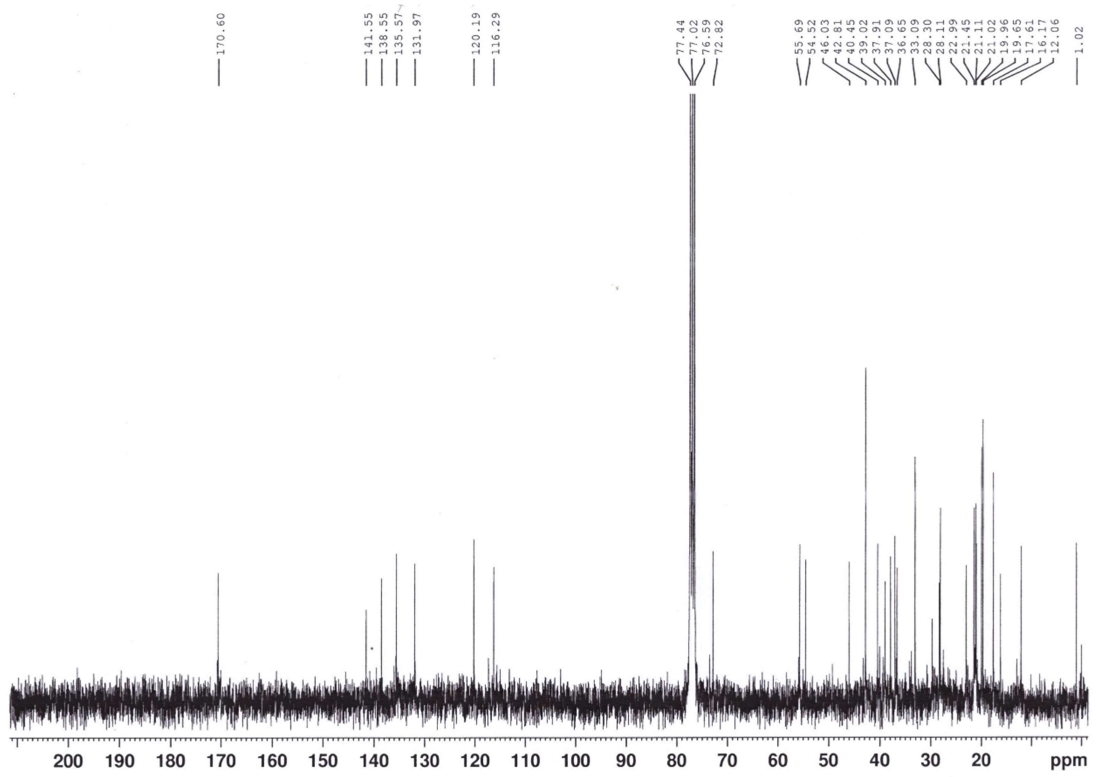

Figure S6. ^{1}H NMR spectrum of (3*R*)-3-(1I-indol-3-ylmethyl)-3, 4-dihydro-1I-1,4-benzodiazepine-2,5-dione (**3**) ($DMSO_{d6}$, 500 MHz).

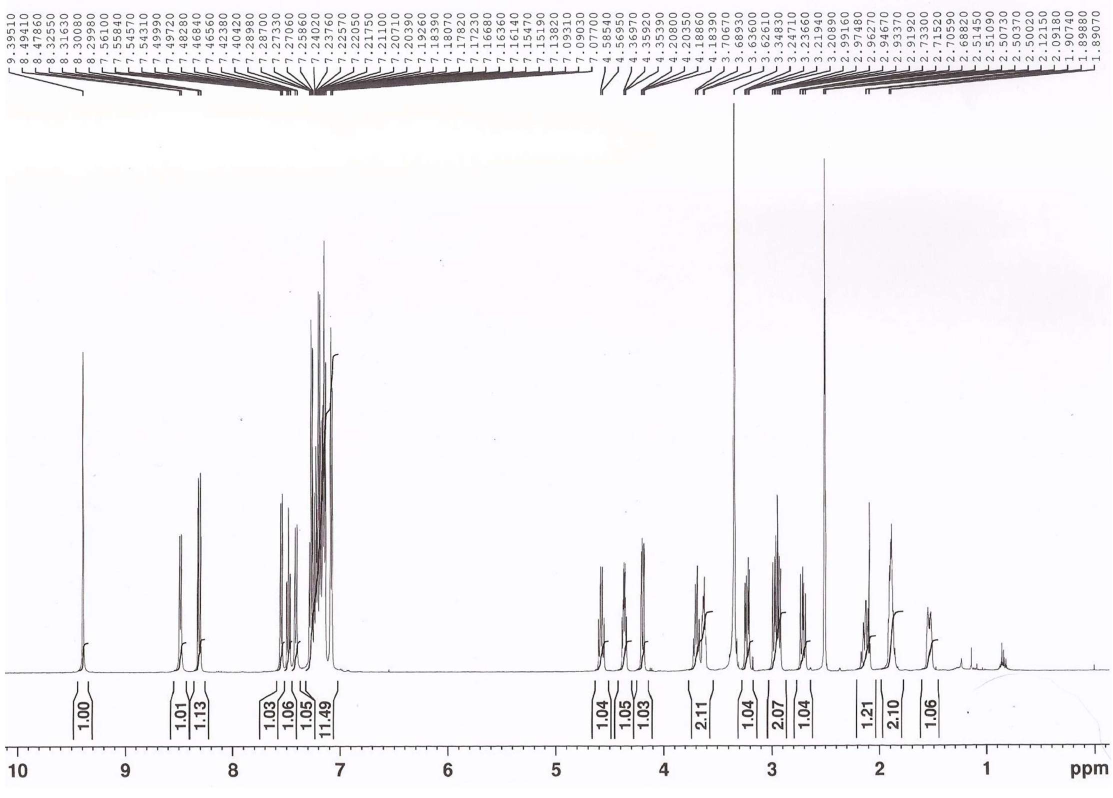

Figure S7. ^{13}C NMR spectrum of (3*R*)-3-(1I-indol-3-ylmethyl)-3, 4-dihydro-1I-1,4-benzodiazepine-2,5-dione (**3**) (DMSO$_{d6}$, 125 MHz)

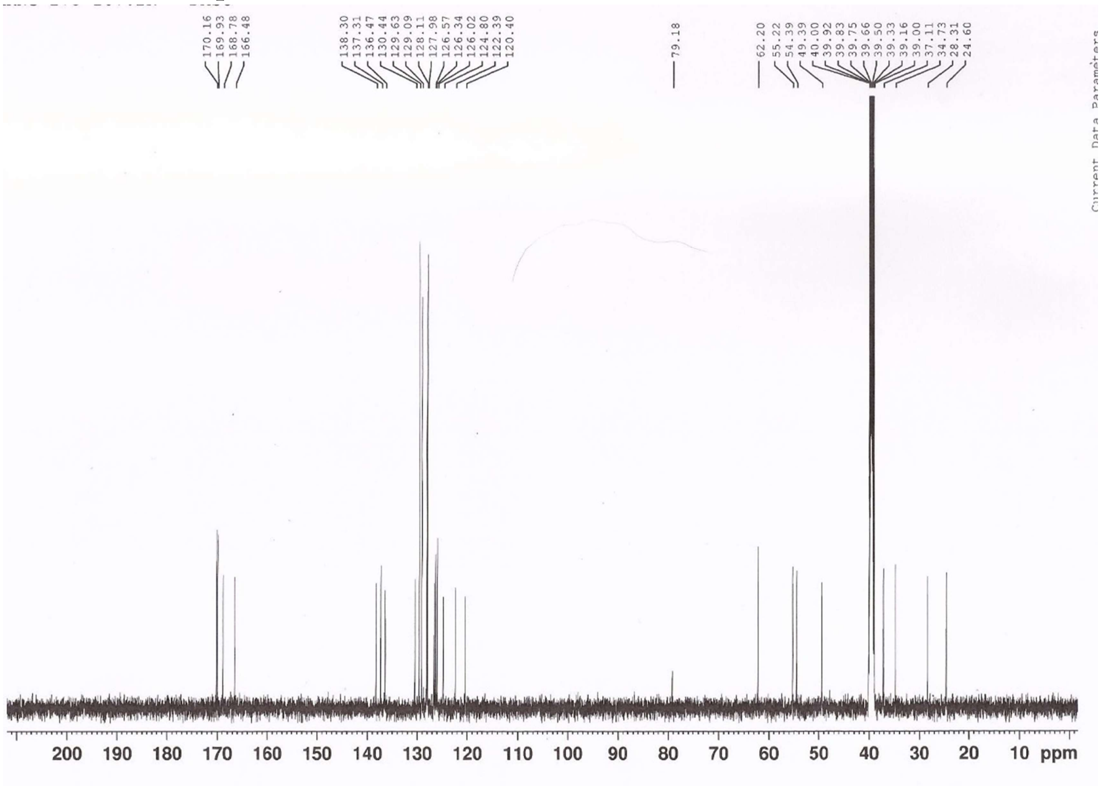

Figure S8. ^{1}H NMR spectrum of hydroxytakakiamide (**4**) (CDCl$_3$, 500 MHz).

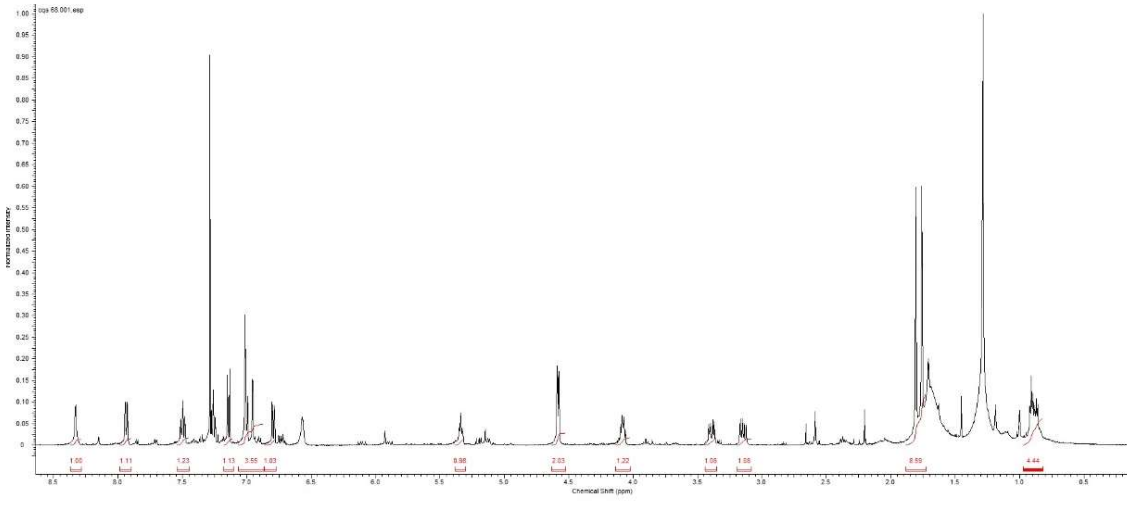

Figure S9. ^{13}C NMR spectrum of hydroxytakakiamide (**4**) ($CDCl_3$, 125 MHz).

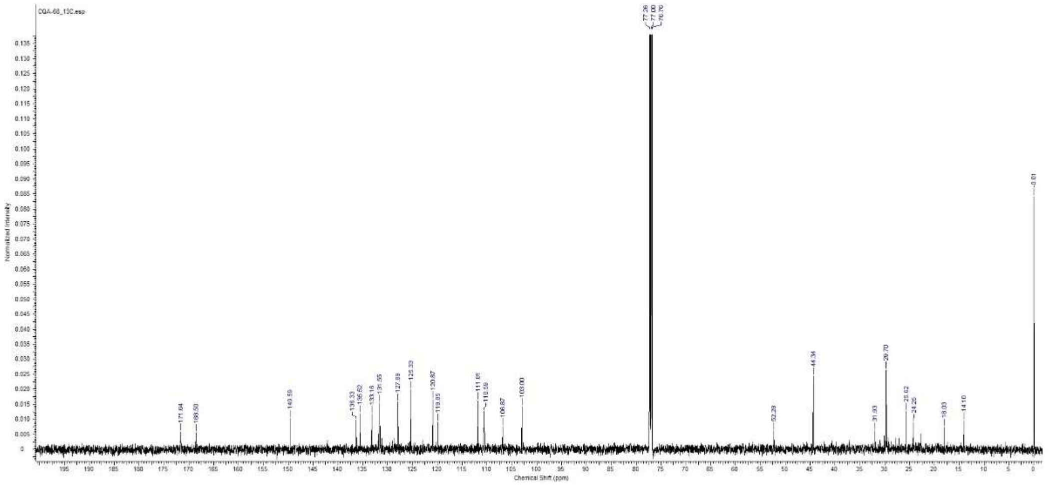

Figure S10. COSY spectrum of hydroxytakakiamide (**4**) ($CDCl_3$, 500 MHz).

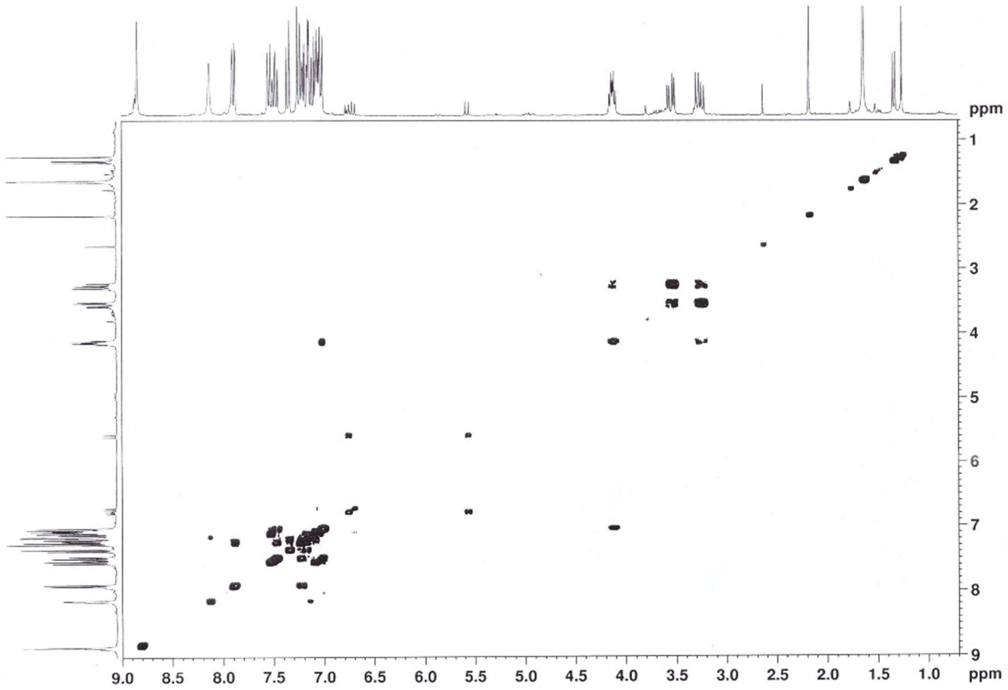

Figure S11. HSQC spectrum of hydroxytakakiamide (**4**) ($CDCl_3$, 500 MHz).

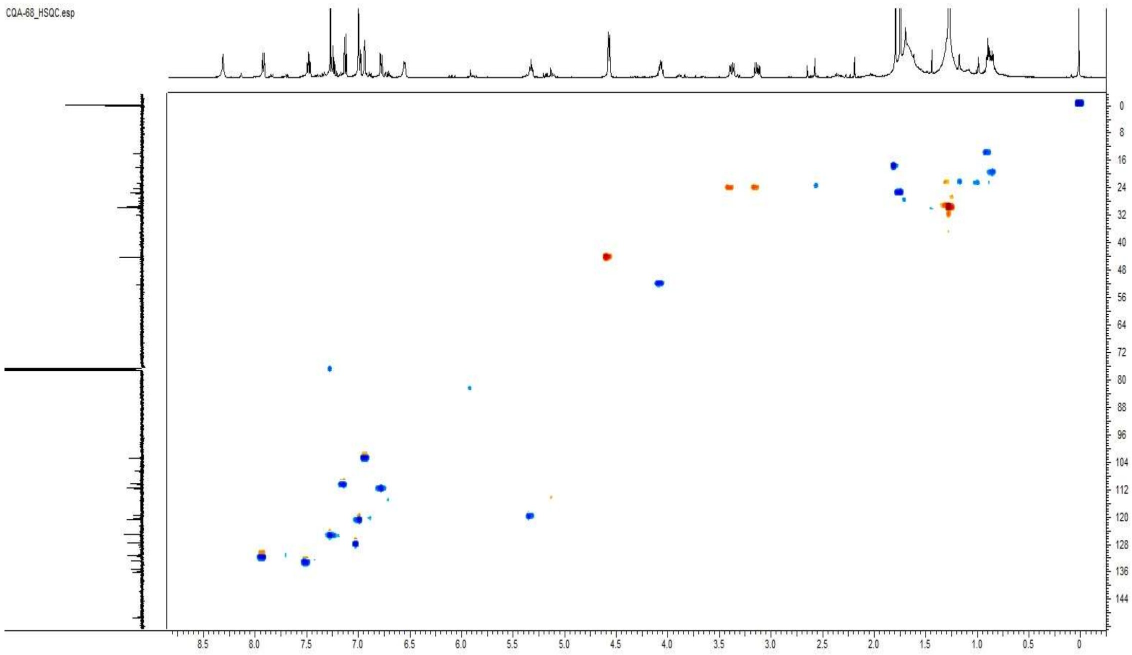

Figure S12. HMBC spectrum of hydroxytakakiamide (**4**) ($CDCl_3$, 500 MHz).

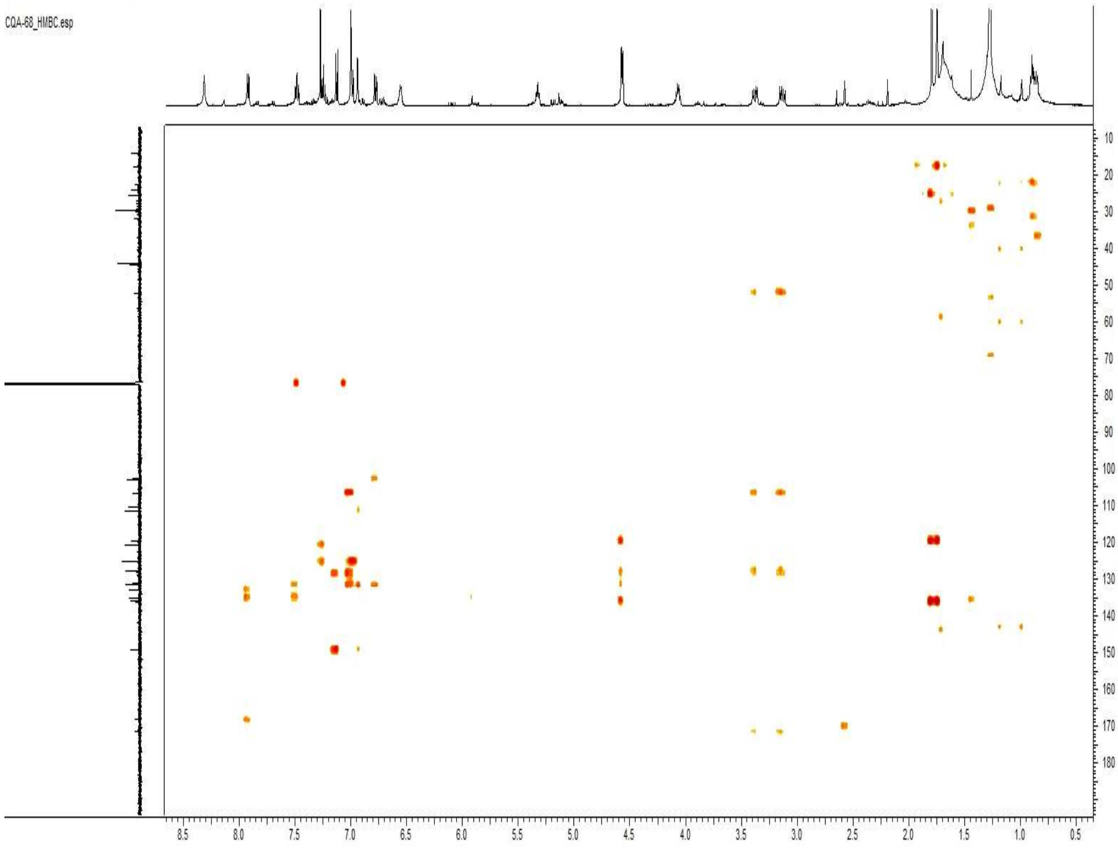

Figure S13. (-)-HRMS spectrum of hydroxytakakiamide (**4**)

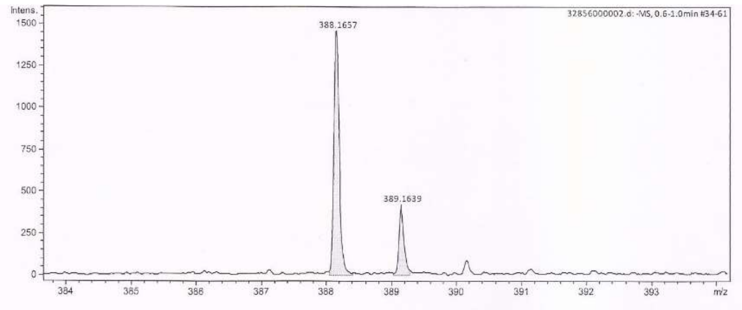

Figure S14. ^{1}H NMR spectrum of helvolic acid (**5**) ($CDCl_3$, 300 MHz).

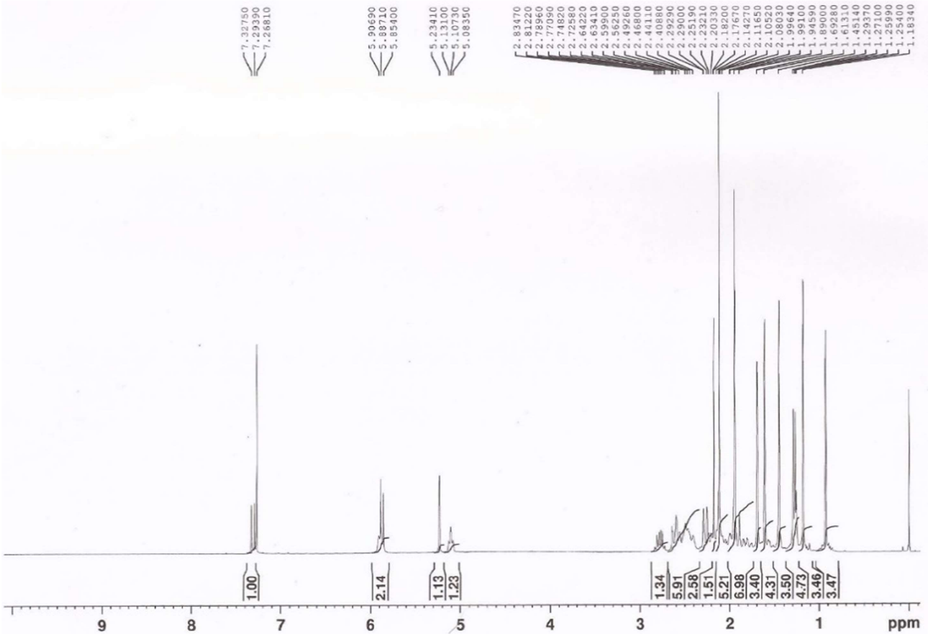

Figure S15. ^{13}C NMR spectrum of helvolic acid (**5**) ($CDCl_3$, 75 MHz).

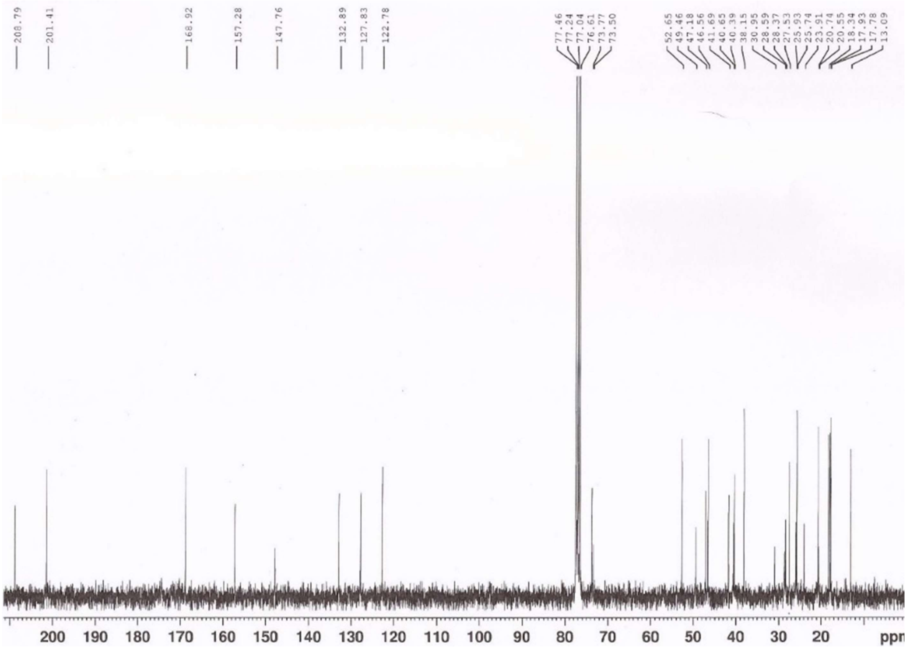

Supplement: Supplementary file 1 [file marinedrugs-22-00097-s001.zip › marinedrugs-2867577-supplementary.pdf]
